# Rates of HBV, HCV, HDV and HIV type 1 among pregnant women and HIV type 1 drug resistance-associated mutations in breastfeeding women on antiretroviral therapy

**DOI:** 10.1186/s12884-018-2120-7

**Published:** 2018-12-22

**Authors:** Judith N. Torimiro, Aubin Nanfack, William Takang, Claude Kalla Keou, Awum Nchenda Joyce, Kevin Njefi, Kimbong Agyingi, Irenee Domkam, Desire Takou, Sylvie Moudourou, Samuel Sosso, Robinson E. Mbu

**Affiliations:** 1 Chantal Biya International Reference Centre for Research on Prevention and Management of HIV/AIDS (CIRCB), Molecular Biology Laboratory, B.P. 3077, Messa, Yaounde, Cameroon; 2 Chantal Biya International Reference Centre for Research on Prevention and Management of HIV/AIDS (CIRCB), Laboratory of Immunology and Microbiology, Yaounde, Cameroon; 3 Faculty of Health Sciences, University of Bamenda, Bamenda, Cameroon; 4 Faculty of Medicine and Biomedical Sciences, University of Yaounde I, Yaounde, Cameroon; 5 Faculty of Health Sciences, University of Montagnes, Bangangte, Cameroon; 6 Chantal Biya International Reference Centre for Research on Prevention and Management of HIV/AIDS (CIRCB), Data Analysis and Impact Studies Unit, Yaounde, Cameroon; 7 Chantal Biya International Reference Centre for Research on Prevention and Management of HIV/AIDS (CIRCB), Medical Unit, Yaounde, Cameroon; 8 Chantal Biya International Reference Centre for Research on Prevention and Management of HIV/AIDS (CIRCB), Clinical Diagnostics Laboratory, Yaounde, Cameroon

**Keywords:** HBV, HCV, HDV, HIV, Mother, Child, Transmission, Vaccination, PMTCT, Drug resistance

## Abstract

**Background:**

HBV, HCV, HDV and HIV are blood borne and can be transmitted from mother-to-child. Reports of HBV infection rates show up to 11.9% in Cameroon while for HCV, the rate is less than 2%. More so, as pregnant women get enrolled in the HIV PMTCT Programme and stay in the care continuum, selection of HIV-1 drug resistant strains is evident. We sought to determine the seroprevalence of HBV, HCV, HDV and HIV among pregnant women, assess their knowledge, attitudes and practices on transmission and prevention of HBV infection, and determine HIV drug resistance profile of breastfeeding women.

**Methods:**

A serosurvey of HBV, HCV, HDV and HIV was carried out among 1005 pregnant women in Yaounde, Cameroon. In 40 HIV-infected breastfeeding women enrolled in the PMTCT Programme, HIV-1 genotypes and HIV-1 resistance to NRTIs, NNRTIs and PIs, were determined by phylogeny and the Stanford University HIV Drug Resistance interpretation tool, respectively.

**Results:**

Among the pregnant women, the rates of HIV-1, HBV, HCV and HDV infections were 8.5, 6.4, 0.8 and 4.0%, respectively. About 5.9% of the women knew their HBV status before pregnancy unlike 63.7% who knew their HIV status. Although 83.3% reported that vaccination against HBV infection is a method of prevention, and 47.1% knew that HBV could be transmitted from mother-to-child, only 2.5% had received the Hepatitis B vaccine. Of the 40 women on antiretroviral therapy (ART), 9 had at least one major resistance-associated mutation (RAM, 22.5%) to NRTI, NNRTI or PI. Of these M184 V (12.5%), K70R (10.0%), K103 N (12.5%), Y181C (10.0%), M46 L (2.5%) and L90 M (2.5%) were most frequently identified, suggesting resistance to lamivudine, nevirapine, efavirenz and zidovudine. Eighty four percent were infected with HIV-1 recombinant strains with CRF02_AG predominating (50%).

**Conclusions:**

The rates of HBV and HIV-1 infections point to the need for early diagnosis of these viruses during pregnancy and referral to care services in order to minimize the risk of MTCT. Furthermore, our results would be useful for evaluating the HIV PMTCT Programme and Treatment Guidelines for Cameroon.

**Electronic supplementary material:**

The online version of this article (10.1186/s12884-018-2120-7) contains supplementary material, which is available to authorized users.

## Background

Hepatitis B Virus (HBV), Hepatitis C Virus (HCV), Hepatitis delta virus (HDV) and Human Immunodeficiency Virus Type 1 (HIV-1) are blood borne and can be transmitted from mother to child. Africa bears about two-thirds of the global HIV burden [1] with women of child-bearing age at higher risk of infection. While the tide of HIV infection is increasing in many countries in sub Saharan africa, Cameroon records a rate of 4.1% in 2015, and HBV infection rate of 4.3 to 11.9% [2, 3] and 28% of HBeAg [2] among pregnant women. Children born of HBeAg-positive mothers have a 45.5% chance of infection if no prevention intervention is offered [4] while only 10.2% may get infected if cesarean section is offered and 28.0% if by vaginal delivery [5]. We however lack accurate and recent epidemiologic data on HCV infection among pregnant women in Cameroon although low rates have been reported in the early 2000. Meanwhile, HCV RNA-positivity has been associated with the risk of vertical transmission, contributing to a rate of transmission of 4 to 5%. The risk becomes greater if the mother is co-infected with HIV. On the other hand, HDV infects 15 to 20 million people worldwide. In a rural District in northern Cameroon, anti-HDV prevalence of 7.3% among pregnant women was reported [6] and among adults in Yaounde, 22.7% [7].

Despite this burden and available information on modes of transmission and prevention of these viruses, prevention and control efforts have not been optimal in low- and middle- income countries (LMIC). To promote the WHO initiative to eliminate viral hepatitis by 2030, the incorporation of other communicable diseases in the ongoing HIV and Syphilis Prevention of Mother-to-child Transmission (PMTCT) Programmes is imperative [8]. The HIV PMTCT Programme is integrated into the Mother-Newborn and Infant Service in Cameroon, with coverage from 68 to 92%, while the National Early Infant Diagnosis (EID) is at 48%. In spite of the services available for pregnant women at the antenatal clinic (ANC), first antenatal consultation was at 76% in 2016 with 88% of the women who knew their HIV status and of whom 8% were tested at the time of labour.

The AIDS Treatment Guidelines for Cameroon do not incorporate HIV-1 drug resistance (DR) testing at initiation of ART for the pregnant women nor the infants enrolled in the PMTCT Programme. Therefore, the impact of ART in pregnancy and on future therapeutic response of the mother or infant, has not been fully studied. In 2016, 75.7% of pregnant women were on antiretroviral therapy (68.9% of the Centre Region where Yaoundé is situated). In spite of these interventions, a rate of 5.6% of vertical transmission was reported with mother and infant receiving antiretroviral (ARV) drugs [9].

Resistance to nucleoside and nucleotide reverse transcriptase inhibitors (NRTIs) and non-nucleoside reverse transcriptase inhibitors (NNRTIs) can occur in infants who become infected with HIV in spite the ARV-based interventions. In the KiBS study carried out in Kenya, 67% of infants who were infected despite maternal triple prophylaxis, had drug resistant HIV-1 strains, while in Cameroon a rate of transmitted drug resistance (TDR) of 7% in treatment-naïve pregnant women was reported [10].

The scope of HBV, HCV, HDV or HIV infection in pregnant women needs to be better understood for resources to be allocated to scale-up the PMTCT Programme and improve the quality of life of infants exposed and/or infected with these viruses. The aims of this study were therefore to determine the seroprevalence of HBV, HCV, HDV and HIV, assess knowledge and attitudes of pregnant women on HBV transmission and prevention, and determine the profile of HIV-1 drug resistance among breastfeeding women who were involved in the PMTCT Programme in Yaoundé, the capital city of Cameroon.

## Methods

Ethical approval to carry out these studies were obtained from the Chantal Biya International Reference Centre for Research on Prevention and Management of HIV/AIDS (CIRCB) Institutional Review Board (IRB).

### Selection of study sites and study participants

From September 2011 to April 2015, we conducted a cross-sectional study in the antenatal consultation Departments of three tertiary hospitals in Yaoundé, Cameroon. Two groups of women were recruited for these sub studies.

*Virus Seroprevalence, and Knowledge, Attitude and Practice (KAP) Survey among Pregnant Women sub Study (Group 1):* we used a formula to estimate a population proportion with specified absolute precision. The confidence level was 95% and a prevalence of 2.5% of HCV (which is the least compared to the HIV-1, HBsAg or HDV) were used to calculate the sample size of pregnant women. With an absolute precision of 1%, we calculated a sample size of 937 and added 10% for missing data, giving a final sample size of 1030 of women of age above 21 years. We however, recruited 1005 within the study period.

*HIV Drug Resistance among Breastfeeding Women on Antiretroviral Therapy (ART) sub Study (Group 2):* out of 85 HIV-infected women detected in the Group 1 sub study, 50 were enrolled in the PMTCT Programme. They were HBV-negative, HCV-negative, and breastfeeding (for at least 6 weeks) and on ART. Of these, 40 samples were successfully sequenced for prediction of HIV-1 drug resistance or susceptibility.

### Data collection and analysis

Following a written informed consent, a questionnaire (Additional file 1) was used to collect demographic data, knowledge, attitude, and practices relating to HBV transmission and prevention (Group 1). While in Group 2, data on AIDS treatment history were collected from the participants’ hospital records. The data from the questionnaire were analyzed using the EPI Info 7.2 software. The data was summarized using means and standard deviation for quantitative variables and frequencies for qualitative variables. Associations between qualitative variables were done using Fisher’s Exact test and the significance level was 5%.

### Screening for hepatitis B virus, hepatitis C virus, hepatitis delta virus and HIV infections

HIV-1 antibodies were detected in plasma samples using 2 rapid tests and samples with discordant results were further tested using a fourth generation ELISA (Genscreen ULTRA HIV Ag-Ab). Similarly, the women were screened for HBsAg, HBeAg, anti-HBe, anti-HBc, anti-HCV, anti-HDV by ELISA (Monolisa™). The women diagnosed positive for any of the viruses were interviewed on the awareness of their serological status, and referred to Care services.

### Measurement of T lymphocyte CD4 level, plasma HIV-1 RNA load and determination of HIV-1 drug resistance

T lymphocyte CD4^+^ cell counts were determined by flow cytometry using the BD FACScalibur™ cytometer [11] and plasma HIV-1 RNA load using the Abbott RealTi*m*e HIV-1 assay [12]. To determine specific mutations associated to drug resistance in the protease-reverse transcriptase regions of HIV-1 and genotyping, RNA extraction was done from plasma using QIAamp viral RNA mini kit [13], amplified over a 1197 base pair (bp) fragment and sequenced directly as previously described [14] using the Applied BioSystems Model 3130XL Genetic Analyzer [15].

All nucleotide sequences were automatically aligned with reference sequences of all known HIV-1 Group M subtypes and predominant circulating recombinant forms (CRFs) from the Los Alamos HIV sequence database using CLUSTAL X with minor manual adjustments [16]. Phylogenetic analyses were conducted using the MEGA software package, and trees were constructed by the neighbor-joining method. The protease-reverse transcriptase DNA sequences were analyzed for mutations associated to drug-resistance using the Stanford University HIV Database genotypic resistance interpretation algorithm [17].

## Results

I) **General Characteristics of Study Population**

Overall, 1005 pregnant women (Group 1) were included in this study, of age range from 18 to 43 years (mean age of 27.49 ± 5.18 years). A majority of the women were in the third trimester (69.9%) and the gestational age ranged from 6 to 42 weeks with a mean of 29.49 ± 7.39 weeks. Multigravid women were the most represented (68.3%), and gravidity ranged from 1 to 8 pregnancies (Table 1).

**Table 1:** Demographic data of Pregnant Women (Group 1; *N* = 1005)

Characteristics	Value
Mean age	27.49 ± 5.18 years
Mean gestational age (at 3rd trimester)	29.49 ± 7.39 weeks
Mean gravidity	2.56 ± 1.47 pregnancies
Multigravid	68.3%
Marital status	Frequency (%)
Married	62.1
Cohabitation	20.2
Single	17.8
Educational Level	Frequency (%)
Secondary school level	57.3
Higher level	31.6
Profession	Frequency (%)
Jobless	35.8
Self-employed	18.1
Student	23.3

Over 50% of the HIV-infected women were diagnosed for HIV infection during pregnancy at their visit to the antenatal clinic. Of these, 36% were tested during the last pregnancy and 19% during prior pregnancies while 45% were tested during Voluntary Testing and Counselling Campaigns.

Group 2 consisted of 50 HIV-infected and breastfeeding women on ART of mean age of 30 (± 4 years), with 50% married. Of these 50, only 40 specimens were analyzed for HIV-1 genotypic drug resistance. All the women were on zidovudine plus lamivudine plus nevirapine (AZT + 3TC + NVP) (Table 2).

II) **Knowledge, Attitudes and Practices relating to Hepatitis B Virus Infection**

**Table 2:** Demographic data of HIV-infected and Breastfeeding Women on ART (Group 2; *N* = 40)

Characteristics	Value
Mean age	30 ± 4 years
Marital status	Frequency (%)
Married	50
Educational level	Frequency (%)
Secondary school level	58
Gravidity	
Modal gravidity	2
Modal parity	2
HAART onset	Frequency (%)
Before last pregnancy	62.5
During last pregnancy	17.5
During current pregnancy	20
HAART regimen	
AZT + 3TC + NVP	100

*AZT*: zidovudine

*3TC*: lamivudine

NVP: nevirapine

Among women infected with HBV, 5.9% were aware of their status before pregnancy whereas 63.8% of those infected with HIV had known their status before pregnancy (Table 3). Whereas, among the women co-infected, 75% of them had known only about their HIV serological status and not the other virus. Most of the infected women were single with a high mean gravidity compared to those not infected with any of the viruses (*p* < 0.05).

**Table 3:** KAP Survey on HBV Transmission and Prevention Methods

	Frequency (%)
Awareness of mode of HBV transmission	
Mother-to-child	47.1
Blood transfusion	51.5
Sharing of personal objects	39.5
Sexual intercourse	47.8
Direct contact with body fluid of an infected individual	30.8
Awareness of HBV prevention methods	
Usage of personal objects	37.4
Fidelity	23.7
Abstinence	22.5
Vaccination	83.3
Condom use	30.4
Awareness of virus serologic status before study	
HIV	63.8
HBV	5.9
HCV	0
HBV vaccination	2.5

The most common method of transmission of HBV reported by the women was blood transfusion (51.5%), while 47.1 and 47.8% knew that HBV could also be transmitted from mother-to-child and through sexual intercourse, respectively. More so, 23.7 and 22.5% reported that HBV can be prevented if fidelity is practiced within married couples, and the use of condoms, respectively. Although 83.3% of the women admitted that vaccination was a means of preventing HBV infection, it was found that only 2.5% had received the hepatitis B vaccine (Table 3).

III) **Seroprevalence of HIV-1, HBV, HCV and HDV in Pregnant Women**

Overall, 15.0% (151/1005) of the women were mono-infected for either HIV-1, HBV or HCV. Rate of HCV infection was 0.8%, of HBV (HBsAg+) was 6.4, and 8.5% for HIV-1 (Table 4). No cases of HBV and HCV co-infection were identified while 0.1% (1/1005) was co-infected with HIV-1 and HCV. The age group of 31 to 35 years was most frequently infected with at least one of the viruses (22.8%).

**Table 4:** Seroprevalence of HIV-1, HBV, HCV and HDV among Pregnant Women

		Hospital			Total number of positives	Overall seroprevalence
		Antenatal clinic 1	Antenatal clinic 2	Antenatal clinic 3		
	Total number tested	506	249	250	151^a^	(%)
Rate of biomarker (%)	anti-HIV-1 antibodies	11.1	8.4	3.2	85	**8.5**
	HbsAg	6.3	8.4	4.4	64	**6.4**
	anti-HBs	24.3	23.2	22.4	237	**23.6**
	HBeAg	12.1	11.9	1	82	**8.2**
	anti-Hbe	18.8	16.8	17.6	182	**18.1**
	anti-HBc	37.0	28.4	34.8	347	**34.5**
	anti-HCV	0.4	0.2	0.2	8	**0.8**
	anti-HDV				40	**4.0**

^a^Total number of women infected with HIV, HBV or HCV

Forty of the 64 HBsAg positive women were co-infected with HDV (4.0%) in Group 1. Rates of other HBV infection markers were 23.6% for anti-HBs, 8.2% for HBeAg, 18.1% for anti-HBe and 34.5% for anti-HBc (Table 4).

IV) **HIV Type 1 Genetic Diversity**

**Fig. 1:**
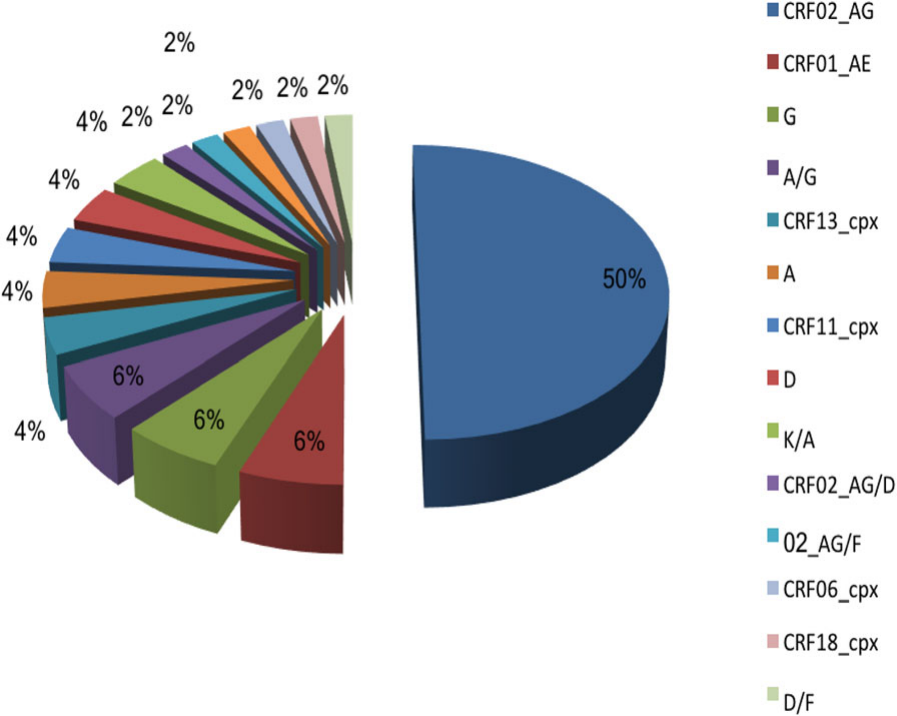
Distribution of HIV-1 Genetic variants. Each colour represents a HIV-1 genetic variant. The pie chart shows the proportion of each HIV-1 genetic variant identified in the study

Analysis of 40 HIV-1 protease-reverse transcriptase sequences revealed a broad genetic diversity dominated by recombinant variants (84%), with the CRF02_AG variant most commonly detected (50%) (Fig. 1). Apart from CRFs and CRF-containing recombinants, recombinants of subtypes along the protease-reverse transcriptase region A/G (6%), D/F (2%) and K/A (4%) were also identified.

V) **Frequency of HIV-1 Drug Resistance-associated Mutations**

Mutations-associated to resistance to NRTI, NNRTI and PI were detected in 9 out of 40 specimens (22.5%) (Table 5).

**Table 5:** Frequency of HIV-1 Mutations associated to Resistance to different Classes of Antiretroviral (ARV) Drugs

Nucleo(s)tide reverse transcriptase inhibitors (NRTI)			Non nucleoside reverse transcriptase inhibitors (NNRTI)			Protease inhibitors (PI)		
Major RAM	Number	Frequency (%)	Major RAM	Number	Frequency (%)	Major RAM	Number	Frequency (%)
M184 V	5	12.5	K103 N	5	12.5	M46 L	1	2.5
K70R	4	10	YI8IC	4	10	L90 M	1	2.5
T215Y	3	7.5	YI88L/H/C	2	5			
V75 M	3	7.5	H221Y	2	5			
T215F	2	5	P225H	1	2.5			
L201 W	2	5	F227 L	1	2.5			
M41 L	2	5	K238 T	1	2.5			
L74 V	1	2.5						
K219Q/E	1	2.5						
K65R	1	2.5						

Some commonly identified RAMs to NRTIs include M184 V (12.5%) and K70R (10%), and to NNRTIs were K103 N (12.5%) and Y181C (10%), and to PIs M46 L (2.5%) and L90 M (2.5%) (Table 5).

Most of the women (62%) had CD4 counts > 350 cells/ul (mean 374+/− 114 cells/mm^3^). HIV-1 plasma RNA viral load of < 1000 copies/ml was reported for 10 participants (25%), while 6 (15%) had greater than 10,000 copies/ml. The median viral load was 4378 copies/ml (IQR: 2036–8268 copies/ml). The greatest proportion of the study subjects (68%) had viral load values between 1000 and 10,000 copies/ml.

## Discussion

Viruses in pregnant women and their impact on the outcome of the pregnancy and infant health have not been well-studied in the era of PMTCT in Cameroon just as in several sub Saharan African countries. We found in this study that 63.8% of the HIV-infected pregnant women knew their status before pregnancy, whilst none of the HCV-infected individuals and 5.9% of the HBsAg-positive, respectively, admitted that they had known their status before pregnancy. On the other hand, we found 8.2% rate of HBeAg, a risk factor of transmission of HBV from mother-to-child [18]. As declared in the Bordeaux Call of April 2018 [19], WHO estimates that 0.3 and 6% of people infected with HBV and HCV, respectively, know their serologic status in Africa. The marked difference in awareness of the serologic status, between HIV-infected pregnant women and those infected with the other viruses highlights the need to promote screening of HBV, HCV and HDV in women.

We report in this study that 47.8% of the pregnant women reported sexual intercourse as a route of HBV transmission while 47.1% reported the vertical transmission route. Although 83.3% of the study population reported that vaccination against hepatitis B would reduce the risk of transmission of HBV, only 2.5% of the women reported haven been vaccinated. The seroprevalence of HIV in our study was 8.5% higher than that reported in the Demographic and Health Survey (DHS) of 7.6% in 2011 [20].

Only one woman was infected with HCV in our study (0.98%), a rate which is low compared to that of HBV or HIV-1, although Ndumbe and colleagues reported a higher rate in Yaounde of 5.5, and 6.0% in a rural community in the South West Region of Cameroon [21]. However, higher prevalence was found among pregnant women in bigger cities like in Benin City in Nigeria of 3.6% [22]. The co-infection rate of HIV/HBV in this study was 0.5%. This was lower when compared to 9.3% reported by Kfutwah et al. among pregnant women in Yaounde with known HIV status [23], and 7.4% in the general population in Cameroon [24].

Antiretroviral drug resistance testing is rarely used to follow-up HIV-infected pregnant women nor infants on ART in Cameroon. A few studies have been carried out in Cameroon to determine the levels of antiretroviral drug resistance in different populations [25–31], but little has been done on HIV-1 infected women during and after pregnancy. Our study was carried out on samples from 40 HIV-infected and breastfeeding women with the goal to evaluate the pattern of ARV drug resistance in women enrolled in the PMTCT Programme and who were breastfeeding.

The prevalence of antiretroviral drug resistance in this study was 22.5% (9/40). This is higher than the findings of Boghuma and colleagues [32] who evaluated ARV drug resistance in a group of Cameroonian women at 6–8 weeks after delivery and found 4 out of 31 (12.9%) with ARV drug resistant HIV-1. This indicates that ARV drug resistance in women enrolled in the PMTCT Programme in Cameroon may actually be growing following the increasing access to ART. We could however not explain this trend because the ARV drug resistance profile of these women prior to ARV exposure was not known.

Other studies have shown variable levels of drug resistance in HIV-infected women following the PMTCT Programme. The women in this study were exposed to NRTIs and NNRTIs. Of the 40 women, only 6 (15.0%) harboured HIV-1 variants with major mutations associated to NRTI resistance. Of the nine mutations, M184 V (12.5%) and K70R (10.0%) were most frequently identified. The M184 V is known to confer high levels of resistance to lamivudine (3TC) and emtricitabine (FTC), while K70R is known to confer intermediate levels of resistance to zidovudine (AZT). The high frequency of M184 V found in our study could be explained by the fact that lamivudine and zidovudine are part of PMTCT regimens in Cameroon. Meanwhile, 5 women out of 40, harboured mutations that confer resistance to NNRTIs of which K103 N (12.5%) and Y181C (10.0%) were the most frequent. Of the 9 RAMs identified, K103 N was identified in 5 women, which predicts resistance to nevirapine. Y181C as well as K103 N confer intermediate to high level resistance to nevirapine and efavirenz, two NNRTIs commonly used in Cameroon.

On the other hand, two women were found with RAMs to protease inhibitors which were M46 L (2.5%) and L90 M (2.5%). The frequency of PI mutations unlike that of NRTI and NNRTI was low in our study. This could be explained by the fact that PIs are rarely used in Cameroon, and are reserved for patients failing first line regimens which contain two NRTIs and one NNRTI. None of the participants in our study had been exposed to PI, therefore, this could have been a case of transmitted PI resistance. We report a broad genetic diversity in our study dominated by recombinant variants (84%). The most represented HIV-1 subtype in our study population was CRF02_AG (50%), similar to the findings reported from other studies [14, 28, 31]. However, we could not determine the HBV, HCV and HDV genetic variants as well as the antiviral drug resistance pattern due to limited resources. Furthermore, we could not follow-up the infants born to virus-positive mothers to determine the rates of vertical transmission.

## Conclusions

The rates of HBV and HIV-1 infection among pregnant women indicate that if effective prevention interventions are not offered, and sensitization of pregnant women on vertically transmissible viruses is not promoted, then we cannot fully protect the next generation of children from these infections. The frequency of HIV-1 drug resistance is high among breastfeeding women after 6 weeks postpartum. Therefore, drug resistance testing should be used to guide the choice of ART for infants who become infected by their mothers enrolled in the PMTCT Programme. The missing link therefore is the policy of screening pregnant women for other communicable diseases that can be transmitted from mother-to-child, and expand the PMTCT Programme in Cameroon. Bridging this gap is a priority and contribution to the WHO Hepatitis Elimination Initiative in a LMIC.

## Additional file

Additional file 1: Questionnaire, KAP Survey among pregnant women on transmission and prevention of Hepatitis B virus. (DOCX 37 kb)

## Abbreviations

3TC: Lamivudine
AIDS: Acquired Immunodeficiency Syndrome
ANC: Antenatal Care
Anti HBC: Anti Hepatitis B core antibodies
ART: Antiretroviral treatment
AZT: Zidovudine
CD4: Cluster of Differentiation
CRF: Circulating Recombinant Form
DHS: Demographic and Health Survey
DNA: Deoxyribonucleic acid
EID: Early Infant Diagnosis
ELISA: Enzyme linked Immunosorbent Assay
FTC: Emtricitabine
HBeAg: Hepatitis B envelope antigen
HBsAg: Hepatitis B surface Antigen
HBV: Hepatitis B Virus
HCV: Hepatitis C Virus
HDV: Hepatitis D Virus
HIV: Human Immunodeficiency Virus
IRB: Institutional Review Board
LMIC: Low- and Middle- Income Countries
NACP: National AIDS Control Programme
NNRTI: Non nucleoside reverse transcriptase inhibitors
NRTI: Nucleoside or nucleotide reverse transcriptase inhibitors
NVP: Nevirapine
PI: Protease Inhibitors
PMTC: Prevention of Mother to Child Transmission
RNA: Ribonucleic acid
STD: Sexually transmitted diseases
