# Supplementary material for: Rates of HBV, HCV, HDV and HIV type 1 among pregnant women and HIV type 1 drug resistance-associated mutations in breastfeeding women on antiretroviral therapy
Source: BMC Pregnancy Childbirth. 2018 Dec 22;18:504. doi: 10.1186/s12884-018-2120-7 (PMC6303885; doi:10.1186/s12884-018-2120-7)
Supplement: Supplementary file 1 — Questionnaire, KAP Survey among pregnant women on transmission and prevention of Hepatitis B virus. (DOCX 37 kb) [file 12884_2018_2120_MOESM1_ESM.docx]

**QUESTIONNAIRE**

**Knowledge, Attitude and Practice (KAP) Survey on HBV Transmission and Prevention**

**Name of Hospital: Date:**

**Code: Name of participant:**

**Informed Consent obtained** YES NO

1. Age: ……………... years
2. Marital status: Married Single Widowed

Separated Co-habit

1. Educational Level: Primary Secondary Tertiary Uneducated
2. Occupation/profession:
3. Do you know if you have one of these diseases? HIV/AIDS, hepatitis B, hepatitis C, hepatitis delta (circle the correct answer).
4. If your answer to Question 6 is YES, do you know the date you were diagnosed for the disease(s)?

Date of diagnosis: Hepatitis B: HIV:

Hepatitis C: Hepatitis D:

1. Circumstance(s) of diagnosis: (a) personal request (b) health campaign (c) doctor’s advise (d) other (specify)
2. Do you know how hepatitis B can be transmitted? Yes No

If Yes, name the different ways that hepatitis B can be transmitted.

1. Do you know how hepatitis B can be prevented?

If Yes, name different ways of preventing hepatitis B.

1. Have you been vaccinated against Hepatitis B: Yes No

If YES, date of vaccination:

1. How many times have been pregnant?
2. Contacts:

Next of Kin 1 (name/contact):

Next of Kin 2 (name/contact):

Name of Consulting Physician:
